# The effects of floor plan representations on preferences for apartments

**DOI:** 10.1007/s10901-022-09966-w

**Published:** 2022-08-27

**Authors:** Jacqueline Baker, Harmen Oppewal

**Affiliations:** Department of Marketing, Monash University, 900 Dandenong Road, Caulfield East, Monash, VIC 3145 Australia

**Keywords:** Housing preference, Representation format, Floor plan layout, Tenant preferences, Build-to-rent, Apartments, Lay-individuals, Stated preferences

## Abstract

In the real-estate industry, floor plans are commonly used to communicate spatial layouts of housing alternatives to house hunters. Using the method of stated preferences, this research investigates whether lay-individuals’ preferences for layout attributes differ when floor plans versus text descriptions are used to measure preferences for build-to-rent apartments. The study involved 417 student respondents evaluating four hypothetical apartments twice, with the apartments varying in two focal attributes, layout orientation, and dining space status. Findings from the experiment indicate that floor plan representations of apartments are rated higher overall than text representations; however they also suggest that the effects of the two focal attributes on apartment preferences are larger for text than floor plan formats. Further, effects of the attributes on apartment preference ratings are shown to depend on the participants’ user goal and on their level of attribute knowledge. The main contribution of this research is that it demonstrates how representation format influences housing preferences, and further, how lay-individuals’ judgements of layout attributes depend on the individual’s goals and their knowledge of the attributes. These findings are relevant for future stated preference studies and for real estate agents and property developers when considering what information to provide to prospective buyers and renters of sight-unseen residential property.

## Introduction

Floor plans are widely used in the real-estate industry to communicate the design and layout of spaces, assisting prospective buyers and renters with piecing design features together, imagining what living in the property would feel like and recalling property post inspection (Ewenstein & Whyte, 2009; Gross, 1996; Milliken, 2015). However, floor plans can be problematic for lay-individuals. While experts are trained in reading and visualising floor plan layouts, without the benefit of training, lay-individuals may attend to irrelevant variables (Ishikawa et al., 2011) and the complexity of layout formats may be too great for them to fully comprehend their implications. It is not surprising that off-the-plan buyers have reported, upon seeing their apartment for the first time, that they are nothing like they imagined (McIntyre, 2013; Williams, 2015).

Lay-individuals’ preferences for residential environments and related products and services is a topic spanning decades of research in the housing and stated preference literature (Hunt, 2001; Katoshevski & Timmermans, 2001; Oppewal et al., 2005, Orzechowski et al., 2005, 2012; Verhetsel et al., 2017; Walker et al., 2002; Wang & Li, 2004). The measurement of such preferences, ranging from preferences for housing of experts such as real-estate agents, valuers and architects, to preferences of lay-individuals is well documented in the literature (Antoniou & Dimopoulos, 2018; Boumová & Zdráhalová, 2016; Gifford et al., 2000, 2002; Llinares & Iñarra, 2014).

However, the effect of floor plans, as a form of visual format, on preference measurement relative to traditionally used verbal formats of multi-attribute housing alternatives is yet to be examined. Several scholars have experimented with using visual formats to simulate real-world housing-related settings such as immersive virtual reality (e.g. Azmi et al., 2021; Birenboim et al., 2019), 360° walk-throughs (e.g. Orzechowski et al., 2012; Pleyers & Poncin, 2020), photographs (e.g. Jansen et al., 2009), renderings (e.g. Acemyan & Kortum 2018) and floorplans (e.g. Gao et al., 2013). Some of these studies have compared effects between visual and verbal formats and found similar or higher preference ratings for visual formats demonstrating that visual formats used interchangeably or together with verbal formats potentially improves the validity of preference measurements (e.g. Jansen et al., 2009; Orzechowski et al., 2005; Patterson et al., 2017).

Without comparing their effects, it remains unknown whether preference measurements yield similar results for both formats, bringing into question whether or to what extent floor plans can be used interchangeably, or in combination with traditional verbal formats. The aim of this study is therefore to measure and compare preferences for layout attributes as measured using traditional verbal formats and floor plan formats and thus assess the role of floor plans as representations of sight-unseen apartments in the evaluation of architectural layout preferences. We develop an application that measures ratings for hypothetical apartments in experimentally designed tasks as used in stated preference (SP) measurement methods (Louviere, 1988a; Louviere et al., 2000) and, using student samples, we investigate whether lay-individuals’ stated preferences for apartment layout attributes differ when floor plans versus text descriptions are used to measure apartment preferences.

Establishing whether floor plans have a role in describing layouts more realistically than verbally described layouts will help determine whether they provide a useful addition to the SP measurement toolkit as well as whether they should be considered when deciding what information to provide to prospective buyers or renters, especially when procuring property sight-unseen such as buying off-the-plan or renting without inspecting the property. These considerations extend also to the context of immersive technologies such as augmented or virtual reality housing applications in terms of determining which information to provide in such applications in order to best assist prospective buyers and renters in understanding the benefits and value of different housing options.

Prior to describing the study details, we first further describe the study context and contribution and review the relevant literature around housing preference and choice, representation format and preferences for floor plans in the context of SP methods. Following this, we present our hypotheses and the conceptual model. The final section of the paper discusses the study’s findings and draws conclusions.

## Research context and contribution

Property developers, landlords, leasing and selling agents typically offer a suite of conventional representations to assist consumers with visualising existing and future (e.g. off-plan) housing alternatives, for example, text descriptions, photographic images, renders, floor plans, location maps, 360° walk-throughs and built showrooms. Due to the impact of the COVID-19 pandemic on physical inspection of property (Sulaiman et al., 2020), and the questionable ability of conventional representations to fill the imagination gap for consumers making purchase or rental decisions about sight-unseen property (Andrew & Larceneux, 2018), scholars are suggesting the property industry requires a digital transformation to better meet time-poor, tech-savvy, pandemic-affected consumer needs to visualise property options, with tools including immersive technologies such as augmented reality (AR), virtual reality (VR) and mixed reality (MR) and unmanned aerial vehicles or drones (Alaseeri et al., 2021; Azmi et al., 2021).

However, floor plans, although a conventional representation format, provide unique information other representations are unable to describe accurately, that transcend basic size and shape attributes, such as layouts, dimensions, spatial adjacencies, access, connection, and orientation. In some property transactions, floor plan information is bound by the terms set out in purchase contracts. For example, in Victoria, Australia, the floor plan forms part of a registered plan of subdivision for off-the-plan purchases and the Property Law Act 1958, Section 9AC (1) and (2) protect the purchaser of off-the-plan property from material changes to the plan of subdivision such as an amendment that materially affects the lot to which the contract relates—for example, changes to the dimensions of the property.

The current study measures the effect of floor plan representations on stated preference measurements using the apartment sub-type of residential property. Methodologically, the study demonstrates how floor plan information can influence SP measurements of layout attributes. As explained in the next sections, the method of stated preferences uses experimentally designed descriptions of *hypothetical* choice options to measure individual preferences. This method is therefore highly relevant for the real estate industry.

Practically, the current study contributes to understanding tenant preferences for residential apartments and is therefore especially relevant to build-to-rent (BTR) landlords. The BTR asset class is well-established in Europe and North America and emerging in other anglophone liberal welfare states (Nethercote, 2020), as the world becomes more urbanised and owning a home becomes more out-of-reach for the Gen Z and Millennial generational cohorts. The BTR industry has reached a relatively mature life cycle phase in the United States, decades ahead of the United Kingdom where this asset class is still in its infancy (Nethercote, 2021; Scalon et al., 2018) and Australia, where the BTR sector is in an embryonic state (Nethercote, 2020; Delahunty, 2022). However, with statistics reporting that around a third of Australian household tenure is made up of renters (Australian Institute of Health and Welfare, 2021), and the pandemic providing a catalyst in accelerating BTR supply (Lenaghan, 2020), property industry experts are seeing the BTR asset class building some momentum in Australia (Delahunty, 2022; Jones Lang LaSalle, 2020), particularly among asset-light consumers such as students and young professional Gen Z and Millennials.

Of relevance to the current study is that BTR apartment complex landlords need to target their product to the asset-light renter demographic (Delahunty, 2022). Further, this industry is gaining traction most notably in the largest Australian cities, Melbourne, and Sydney where land tax breaks have been provided by the NSW and Victorian Governments (Lenaghan, 2020). Also noted is the lack of studies about preferences for BTR apartments in the SP literature. To this end, this study measures layout preferences of hypothetical apartments for students who are imagining renting their first apartment as a young professional, in South Yarra (a suburb popular with young professionals), in Melbourne, Australia.

## Literature review

This section reviews the literature on (1) housing preference and choice, (2) representation format, and (3) preferences for floor plans in the context of SP methods. Prior to reviewing the literature, it is important to define SP methods as this study involves hypothesis testing of SP stimuli and utilises SP methods as a research method.

SP methods are a family of techniques that measure individuals’ preference statements about multi-attribute products and services. Typically, the presented alternatives are hypothetical, which means the options can include products or services that are not currently available in the marketplace (e.g. Green & Srinivasan, 1978; Hensher et al., 2015; Kroes & Sheldon, 1988; Louviere, 1988a). SP methods allow the researcher to estimate preferences for features or attributes of environments by asking individuals to rate experimentally designed multi-attribute alternatives on a preference scale^1^. Attributes are varied in their level of intensity across the experimental design conditions. Alternative ratings are deemed to represent the total utility for each alternative and are then decomposed into part-worth utilities for each attribute level, revealing the attributes’ relative influence on the alternative ratings. SP methods assume that individuals commence with the acquisition of information, researching the available alternatives and trading off the set of attributes most important to them to use for comparison, evaluation and selection of alternatives within the limits of their search (Louviere, 1988a).

### Housing preference and choice

Stated preferences in the context of housing alternatives and related products and services have been the subject of many studies over the past 40 years or so. Whilst examining various theoretical perspectives and methodological problems, much of the housing preference and choice literature contributes applied knowledge to the gamut of owner-occupier and tenant end-user preferences for housing alternatives. Subsets of owner-occupiers’ housing preferences include related and unrelated household groups (Lan, 2011; Molin et al., 1997), seniors (Chiu & Ho, 2006; Tanaś et al., 2019) and young people (Wu, 2010). Owner-occupiers’ preferences have been studied across all dwelling typologies from apartments to free-standing homes in scenarios such as build-to-own housing (Orzechowski et al. 2005, 2012; Syahid et al., 2021), existing housing (Wang & Li, 2004, 2006; Jiang & Chen, 2016) and refurbished housing (Komatsu, 2021).

Several subsets of tenant preference and choice have emerged in the literature. Students have been a popular tenant subset over the past decades (e.g. DeSarbo et al., 2004; Edwards, 2019; Green et al., 1988; Oppewal & Klabbers, 2003; Oppewal et al., 2005, Verhetsel et al., 2017). Recent studies involving preference and choice of tenant subsets include public housing tenants (Walker et al., 2002) public housing migrant tenants (Zhou & Musterd, 2018), shared housing tenants (Kim et al., 2020) and holiday short-stay/holiday tenants (Martín et al., 2018). At the time of writing there are no preference and choice studies that apply to the build-to-rent tenant subset.

### Representation format

SP researchers traditionally use verbal-based stimuli, rather than visual stimuli to represent multi-attribute alternatives. This is mostly for the practical reason that they are quicker and less expensive to administer (Orzechowski et al., 2012). Some scholars are of the opinion that the additional time and expense required to administer visual formats for SP applications is not worthwhile where the effects of visual and verbal stimuli are consistently similar (e.g. Arentze et al., 2003; Louviere et al., 1987). In other words, they argue that if the effects are around the same, visual formats provide no additional value to the research. However, representations are proxies for actual products and services and play an important role in studies evaluating environments (Bateson & Hui, 1992; Kuliga et al., 2015). The look may be accurately representable by proxies; however the feel of spaces, for example the social energy and ambience, is at threat of being lost in representations (Loomis et al., 1999; Ziegler, 2011).

The general assumption that preferences for hypothetical alternatives represent *actual* preferences has been debated by many scholars (Haghani et al., 2021; Lancsar & Swait, 2014; Louviere, 1988b) including residential environment scholars questioning to what extent traditional text-based (verbal) multi-attribute alternatives of hypothetical built environments predict real world settings (Jansen et al., 2009; Morrow-Jones et al., 2004; Orzechowski et al., 2012; Rid & Profeta 2011) or to what extent visual alternatives predict real world settings (e.g. Meißner et al., 2020).

We argue that verbal-based stimuli may not be adequately realistic to sufficiently represent architectural layouts which fundamentally concern the size, shape and position of spatial elements and their relationships (de las Heras et al., 2014) including spatial adjacencies, access, connection and orientation. Linguistic descriptions of spatial relationships are generally more abstract than visual descriptions, and therefore some of the accuracy and detail of spatial information is lost in language, despite the inherent ability of humans to describe spatial observations (Hayward & Tarr, 1995). For example, when describing spatial relationships in language, such as “above”, “under” and “adjacent”, accurate information about the positioning and relationships to other spaces is not adequately captured, information pertinent to evaluation of architectural layouts.

Further, it is thought that in cases where individuals are evaluating hypothetical settings, visual representations may better convey the spatial relationships of the setting than text-based representations (Green & Srinivasan, 1978; Vriens et al., 1998; Wittink et al., 1994). On the other hand, although language is limited in its ability to describe spatial relationships (Hayward & Tarr, 1995), visual depictions are limited in that they can misrepresent the spatial relations and bias perceptions (Crilly et al., 2004; Lurie & Mason, 2007) by focusing attention on non-systematically varied details (Jansen et al., 2009, 2011).

There are many key SP studies that contribute to knowledge about how visual representations perform relative to verbal formats (e.g. Bateman et al., 2009; DeLong et al., 2021; Jaeger et al., 2001; Louviere et al., 1987; Matthews et al., 2017); however surprisingly, only a few scholars have applied and advanced this knowledge in the area of housing preference and choice. In the context of dwelling design, Orzechowski et al. (2005), compared non-immersive virtual reality simulations and text formats, finding preference ratings to be similar yet in the context of choosing future housing typologies, Jansen et al. (2009, 2011) found that photographic images and artists’ impressions in combination with text descriptions, yielded higher preference ratings than text format. In the context of neighbourhood choice, Mostofi Darbani (2014) found that preference ratings using gaming formats were lower than for text formats yet Patterson et al. (2017), compared gaming formats and text formats, finding respondent preferences to be similar.

Another recent housing preference and choice study analyses housing development alternatives comparing two types of visual presentations—3D film sequences and still images (Rid et al., 2018), finding that stated preferences of still images out-perform 3D film sequences; however this study does not compare the two visual formats with traditional verbal formats.

In sum, the findings are inconsistent, however, apart from Mostofi Darbani’s (2014) study using gaming formats, studies to date have found that visual formats either performed equally as well or outperformed verbal formats in rating and choice tasks, although not without issues such as respondents attending to accidental and non-systematically varied attributes (Jansen et al., 2009, 2011). This demonstrates that visual formats used interchangeably or in combination with visual formats potentially assist respondents with visualisation of housing-related alternatives.

### Preferences for floor plans

A review of the literature revealed no previous SP studies on preferences for floor plans. However, a number of other studies emerged as relevant to the current study. Several authors in the literature mention an absence of cognitive studies into floor plan preferences for housing. Ishikawa et al. (2011) addresses this gap in knowledge by examining how individuals read and comprehend floor plans through asking respondents to classify floor plans into 5 separate dimensions relating to number of bedrooms, shape of plan, area of balcony, access to bedrooms and natural lighting. It was found that respondents paid most attention to the number of bedrooms and the shape of the plan, and the importance of the attribute depended on the cognitive style of the individual—participants with a holistic (or global) cognitive style attended to the configuration of the plan as a whole and participants with a serial (or local) cognitive style attended to individual elements and how they interconnected. Classification of cognitive styles as either holistic or serial is well documented in the literature (e.g. Riding 2001). The authors note that future research is required to examine how experts and lay-individuals read and comprehend floor plans.

Building on Ishikawa et al.’s (2011) study, Gao et al. (2013), note a need to study more complex housing types than detached houses and to that end study layout preferences for apartments where variations of shape, size and natural light have large implications on occupants and spatial efficiency is pertinent. The authors asked various family groups, to choose between 7 floor plans presented in sets of two. The results demonstrated that the smaller the dwelling, the more families prioritised housing utility such as a dedicated dining room. For medium sized apartments, families prioritised privacy, orientation, storage and number of rooms. Practically, this study offers detailed knowledge about buyer preferences for floor plans of medium sized apartments for different family groups in Beijing. The study however did not utilise a stated preference approach and did not test the use of floor plan formats in place of traditional verbal stimuli.

Boumová and Zdráhalová (2016), addressed the expert vs. lay knowledge gap identified by Ishikawa et al. (2011), and measured the effect of being an expert versus a lay-individual on floor plan comprehension using the residential images method (Singelenberg et al., 2011). Attributes varied were the configuration or position in the plan of the open plan living/kitchen, bedroom, bathroom, toilet, entrance hall and utility room in three separate plans of the same overall shape and size. The first of the three was a traditional layout where all rooms are accessed from the entrance hall. The second and third were reconfigured versions of the first, differing in how various rooms are accessed. The authors found that the experts (architects) and lay-individuals had two diametrically opposed viewpoints on the preferred floor plan and explained that architects used a broader range of categories to evaluate layouts than lay-individuals. Architects prioritised separation of private and public zones and accessing the private zone through the living areas rather than the entrance hall, whereas lay-individuals prioritised direct access to all private and public spaces from the entrance hall. Generally, architects preferred the redesigned layout on account of the traditional layout being dated and lay-individuals preferred the original (traditional) layout on account of its usability and practicality.

Montañana and Llinares’ (2015) study, focuses on property developers’ presentation of real estate products in an online setting, building on Llinares and Page’s (2007) study, that analysed consumers’ emotional response to housing promotions. They note that especially in the case of off-the-plan property, which is sold sight unseen, developers need to capture the attention of potential buyers and incite feelings and emotions that influence purchase decisions. The authors found that promotions that highlight layout features such as spaciousness, separation of private and family spaces, the degree of detail and the graphic form of the presentation all positively influence feelings and emotions that affect purchase decisions.

Importantly, it is noted that none of these floor plan preference studies are SP studies. An opportunity exists to test floor plan formats where attribute measurement is concerned with architectural design and layouts. Jansen et al. (2009), Morrow-Jones et al. (2004), Oppewal and Klabbers (2003) and Orzechowski et al. (2005, 2012), collectively note that representation of spatial attributes such as building layout attributes are difficult to represent verbally, highlighting a clear gap in the literature. Without such a study, it remains unknown how floor plans perform compared to verbal formats and whether or to what extent they assist lay-individuals in visualising apartment layouts has not been established. This study seeks to address this gap in knowledge.

## Hypothesis development and conceptual model

As established, floor plans are useful tools for communicating the design and layout of property options to house hunters (Milliken, 2015). Many studies have found that map-like diagrams help individuals to comprehend attributes in their spatial context by assisting them to form mental models that are particularly effective when integrated with text (Butcher, 2006; Glenberg & Langston, 1992; Hegarty & Just, 1993; Langston et al., 1998). However, as the research suggests, without experience or training, lay-individuals are limited in their ability to read and comprehend floor plans (Ishikawa et al., 2011) and therefore likely less responsive to variations in layout attributes in an SP experiment compared to when verbal information is used to describe attributes. We therefore posit that when using text-based formats to imagine building layouts, the effect, or relative importance, of layout attributes in determining apartment preference will be greater than when using floor plan-based formats (H1).

When appraising housing layouts, house hunters assess attributes against their goal-related priorities. Kaplan (1983) suggests that assessment of spatial needs is a process of perception, planning and actions, for example when finding a space in a crowded train or judging whether there is sufficient distance and time between passing traffic to safely cross a road on foot. In the same way house hunters have particular goals from which they appraise layouts on their ability to meet those goals. We posit that individuals prioritise attributes related to their goals, articulating their preferences more sensitively for those attributes (Vischer, 1985). We expect that when individuals are focused on finding evidence that their goal is met, they will attempt to read floor plans, paying attention to features that could meet that goal. Accordingly, it is hypothesised that a user’s goal moderates the effect of representation format such that where a user goal is related to a particular layout attribute, that attribute will be prioritised, and the importance of that attribute will increase (H2a) and this increase will be greater in the floor plan format than the text format (H2b).

It is also expected that once respondents learn about the nature of layout attributes, the difference proposed in H1 will reverse and individuals will become more sensitive to variations represented by floor plans than to variations that are verbally represented. It is common practice in stated preference measurement to educate respondents about the attributes and their levels prior to the measurement task, for example through the use of a glossary or a practice task (Orzechowski et al., 2012). This is to familiarise respondents with the nature of the systematically varied attributes, and as a result, will increase awareness and confidence in the attributes. As shown by Orzechowski et al. (2012), attribute familiarisation tasks improve the internal, external, and predictive validities. It is hypothesised therefore that attribute knowledge moderates the effect of representation format such that where individuals become more familiar with a particular attribute, they will prioritise the attribute and the importance of that attribute will increase (H3a). We expect that this increase will be greater in the floor plan format than the text format (H3b) because after learning about the nature of an attribute, individuals will be looking for that attribute when presented with a floor plan and therefore will demonstrate a greater ability to read and comprehend floor plans.

**Fig. 1:**
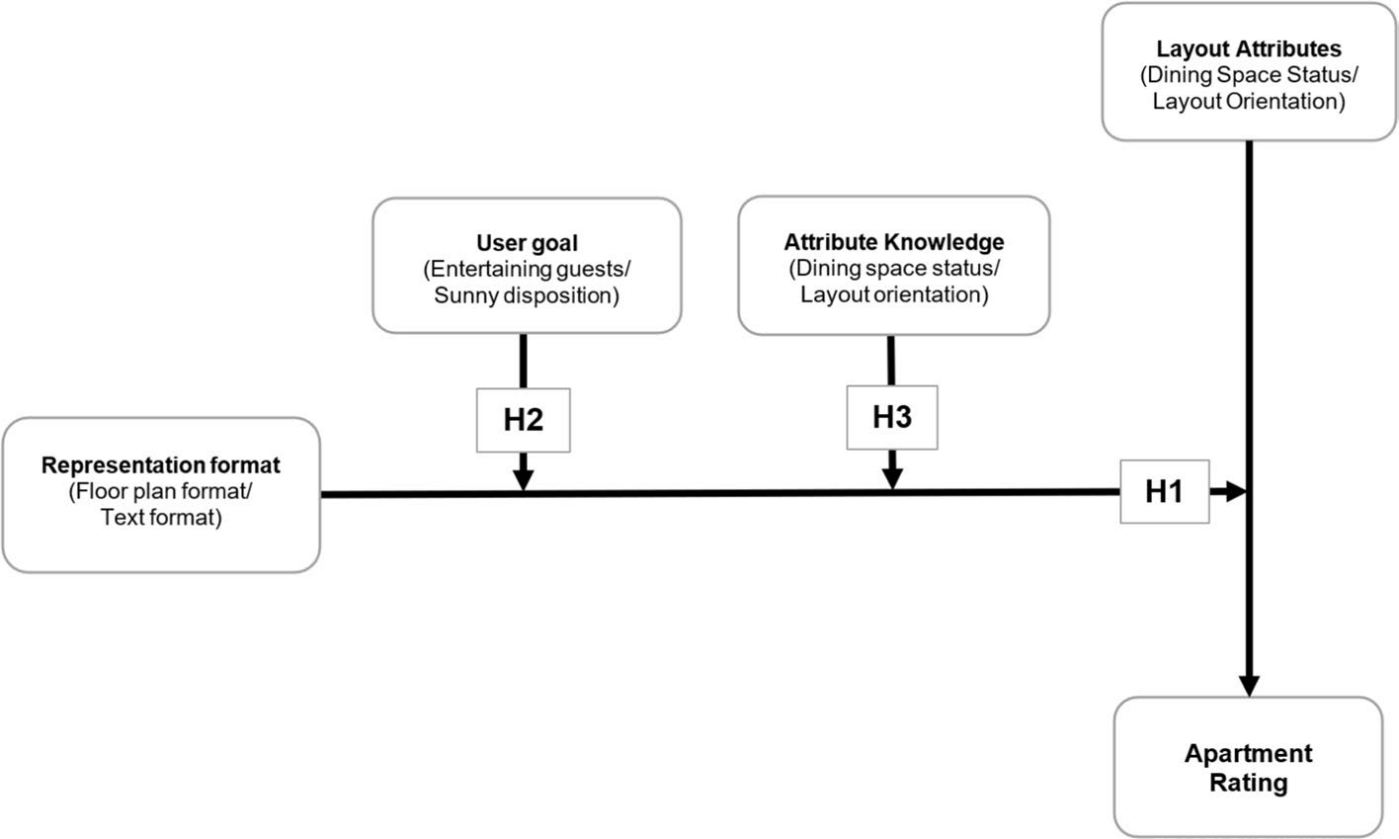
Conceptual model

The conceptual model in Fig. 1 shows how it is proposed that *representation format* moderates the evaluation of two focal attributes on apartment ratings (H1). Further, it shows how *user goal* and *attribute knowledge* moderate the effect of *representation format* on evaluations of the attributes as reflected by *apartment ratings* (H2 and H3, respectively). The next section explains how the theoretical factors in the model were operationalised in the study in terms of attribute and user goal selection. In particular, *dining space status* and *layout orientation* were selected as the focal attributes and user selected user goals are either *entertaining guests* or having a *sunny disposition*.

## Study approach

To test our hypotheses, we selected and varied two attributes that can be accurately described verbally and clearly articulated on floor plans. These were dining space status and layout orientation. Apartments feature dining space status as either non-existent or provided as a dedicated space seating 6 people. Apartments are positioned either to draw indirect or direct sunlight through their large windows, depending on whether they are located on the sun’s path (rising in the east, going around to north and setting in the west—note this study was conducted in the southern hemisphere). We denote the former attribute as *dining space status* (no dedicated dining space vs. dedicated dining space) and the latter as *layout orientation* (north-facing vs. south-facing).

Rather than requesting that participants evaluate these attributes directly, we direct their attention to one of the two attributes in two separate experimental manipulations which we refer to as *user goal* and *attribute knowledge*, respectively. The user goal manipulation asks participants to focus on a goal related to one of the two attributes when rating apartments and the attribute knowledge manipulation asks participants to rate apartments a second time with newly acquired knowledge about one of the two attributes.

We draw on a review of the literature around goal constructs to define user goals as internal representations of desired outcome-related states (Austin & Vancouver, 1996). As noted by Collen and Hoekstra (2001), beliefs, values and goals influence housing choice behaviour. In our study we ask respondents to either prioritise the goal of entertaining guests (by evaluating apartment capacity to hold dinner parties) or having a sunny disposition (by evaluating whether apartments receive plenty of direct sunlight) with the expectation for the former that participants will be focused on dining space status and the latter on the apartment’s orientation to the sun.

To manipulate attribute knowledge, we draw on a study by Orzechowski et al. (2012), in which participants are familiarised with all the attributes used in the experiment by being subjected to a pre-experimental preference training study—the point of difference in this study is that we help participants to learn about the nature of one targeted attribute only, so that we can study the effect of the newly acquired attribute knowledge separately not collectively.

It was expected that the user goal and attribute knowledge manipulations would highlight one attribute over other to prioritise that attribute and that this would influence the variables that participants pay attention to, as reflected in the rating of apartments. In attribute trade-off measurement studies, individuals articulate their preferences more sensitively for attributes that they prioritise (Vischer, 1985). The process guiding participants into prioritising one attribute over the other is achieved through priming, which is defined as the temporary manipulation of the mindset in order to study its passive effects in a subsequent task (Bargh & Chartrand, 2000). Priming works through implicit memory (Schacter, 1992) to temporarily influence a response (Lashley, 1951) working on the premise that prior exposure to a concept increases the probability that it will be used in subsequent tasks (Segal & Cofer, 1960).

## Methods

### Participants

Participants were 417 students (41.6% male) from an Australian university who participated in the study for course credit. Given the participants’ age (84% were between 18 and 20 years old), and their course of study (not design or real-estate related) we assumed they qualified as lay-individuals, not experts. The sample was 65% Australian students and 35% international students. Further, in terms of homogeneity, while there was clearly variation in the sample in terms of participant characteristic as indicated above, given participants were randomly allocated across the different conditions, although it will have affected the statistical power of the study, it can be assumed that this variation did not systematically change the nature of the experimental effects.

### Experimental design

The study used a mixed design consisting of two between-participants factors and two within-participants factors for the testing of H1 and H2. The between-participants component comprised a 2 (representation format: floor plan vs. text) × 2 (user goal: entertaining guests vs. sunny disposition) design. The within-participant factors defined the variations in the two apartment attributes: layout orientation (2: north-facing vs. south-facing) and dining space status (2: dedicated dining space vs. no dining space), which hence resulted in four different apartments.

This design was repeated for the testing of H3, by crossing it with a third between-participant factor: attribute knowledge (2: dining space status vs. layout orientation) and repeating the evaluation of the same four apartments after receiving additional attribute information. In this manipulation, the participant was either given information about how to evaluate dining space status in relation to holding dinner parties or how to evaluate layout orientation in relation to providing natural winter warmth. By repeating the apartment evaluations, the design was extended with one additional within-participant factor (2: apartment rated before vs. after the attribute knowledge manipulation).

In sum, the combinations of the two apartment attributes represented four different apartment alternatives which were shown, in random order, to respondents for consideration and preference articulation in one of two representation formats and for one of two user goal conditions. Once all four alternatives had been presented, participants were exposed to one of two attribute knowledge conditions after which the alternatives were then presented for a second time.

To determine the sample size the original study relied on the rule of thumb that experimental studies typically should have about 50 respondents per between-participant cell, hence 400 was set as the target sample size. Across the design the eight between-participant cells included between 49 and 55 respondents. Power calculations conducted in G-Power for a repeated measures fixed effects Anova for a medium effect size (*f* = 0.25; equivalent to *η*_b_^2^ = 0.06) with 16 groups reveal that with this number of respondents the power exceeds 0.99; when considering a small effect size (*f* = 0.10; equivalent to *η*_b_^2^ = 0.01), the power is 0.66 as calculated by G-Power.

### Measures

Respondents indicated how much they liked each apartment alternative on a 5-point bipolar scale (1 = “dislike extremely”, 2 = “dislike very much”, 3 = “neither like nor dislike”, 4 = “like very much”, 5 = “like extremely”). Further ratings questions were also asked relating to user goal, for example, “how well does the apartment accommodate dinner parties for 6” (relates to entertaining guests) and “what chance does the apartment have of being naturally warm in winter” (relates to sunny disposition), these were also rated on 5-point bipolar scales.

### Procedure

The survey was undertaken on laptops in a behavioural laboratory. Respondents were given an explanatory statement that confirmed the university’s ethics approval and the anonymity of responses and then independently completed the task instructions on each screen. The scenario commenced with a set of instructions: “We are interested in people’s decision-making process when renting apartments”. It asked respondents to imagine that they have graduated and have decided they want to live alone in a 1-bedroom apartment in South Yarra, Melbourne, Australia; and are searching in a price range of $350–$400 per week.

**Fig. 2:**
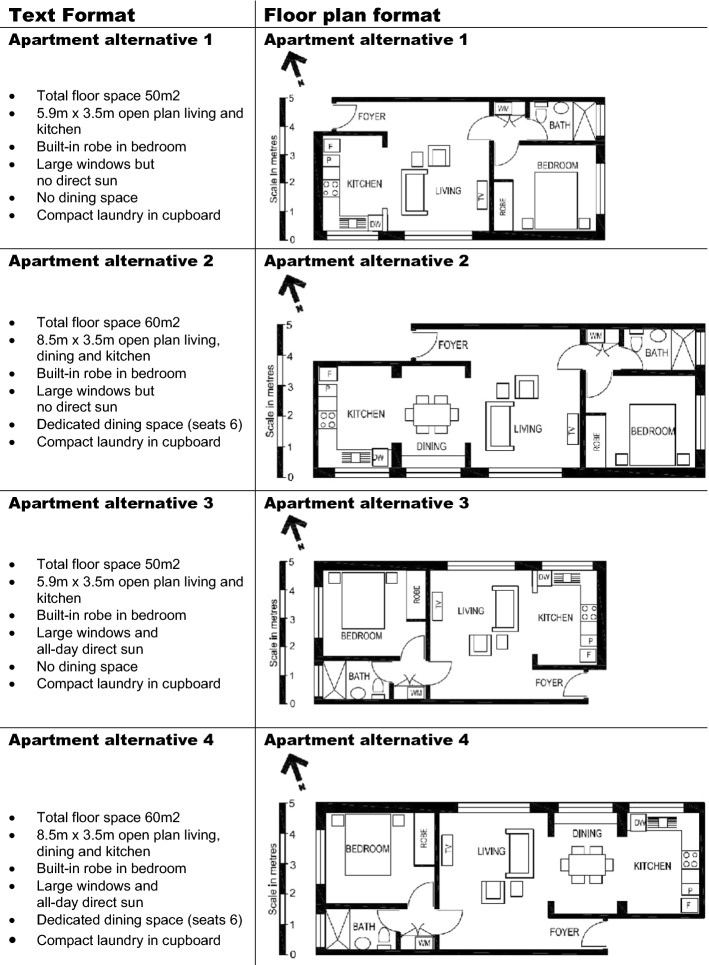
Apartment alternatives

After setting the scenario, participants were given instructions about the user goal to which they were assigned. For the condition “sunny disposition” it was stated; “when considering apartment alternative, you decide whether they can provide natural warmth in winter”. Instructions for the condition “entertaining guests” stated; “when considering apartment alternatives, you decide whether they can accommodate dinner parties for 6 people”. The four apartment alternatives were represented either in text format or floor plan format. Each participant was presented with one format only throughout the survey and asked to evaluate apartment alternatives depending on the user goal condition to which they were randomly assigned. Figure 2 shows all apartment alternatives in both text format and floor plan format.

Respondents assigned with the goal of entertaining guests were asked to appraise apartments on their capacity to accommodate dinner parties for 6 people. Apartment alternatives 2 and 4 shown in Fig. 2 were expected to rate well relative to other alternatives as they have a dedicated dining space that seats 6 guests—in the text formats this is stated explicitly and in the floor plan formats it is articulated with a symbol clearly showing a dining table accompanied by 6 chairs. This is also supported by additional evidence (size of living area) that implies there is sufficient room in the apartment for a dedicated dining space—although we concede that this information may be lost on lay-individuals unfamiliar with average apartment and living room sizes. Apartment alternatives 2 and 4 state that the total floor space is 60 m^2^ and the size of the open plan living, dining and kitchen is 8.5 m × 3.5 m (text format). The floor plan format illustrates the floor space and size of the open plan living, dining and kitchen areas as a scaled version of the apartment layout and via the scale included to the left side of the plan—although this information may be lost on lay-individuals with limited understanding of apartment sizes. In the sunny disposition user goal condition, participants were asked to evaluate what chance apartments had of being naturally warm in winter. Apartment alternatives 3 and 4 were expected to rate well. The text formats state, “large windows and all-day direct sun” and the floor plan formats demonstrate a north-facing orientation and large windows that are located on the sun’s path from morning to late afternoon (rising in the east, going around to north and setting in the west).

Other fixed variables were also included separate from the design. The text format contained fixed variables “built-in robe in bedroom” and “compact laundry in cupboard” which were also fixed in the floor plan with all alternatives of both formats featuring these variables. The function of the non-varying attributes was to prime the respondents to think about and engage with the attributes, as well as strengthening the preference analysis results.

After evaluating the four apartment alternatives, the participant was either given information about evaluating dining space status in relation to holding dinner parties or evaluating layout orientation in relation to providing a sunny disposition. For example, the information about evaluating dining space status read that “assessing whether or not the apartment has enough space for your entertaining needs can be done with the assistance of the scale and by considering the shapes of rooms and how they relate to each other”. The information about evaluating sunny disposition read that “when assessing the position of the property in relation to the sun, check the direction of the north-point adjacent to the floor plan” … and “if the windows of the apartment are located to the east or west of north or directly north, this tells you that the apartment is well positioned in relation to north”.

After the attribute knowledge task, the four apartment alternatives were shown again and one by one rated on the same scale items. The survey concluded with some demographic questions and additional questions that are not further considered in this paper.

## Results

Given the repeated measures nature of the data we used the GLM procedure in SPSS to analyse apartment ratings. Hypotheses 1 and 2 and the manipulations were tested based on the ratings for the four apartment alternatives. Independent variables were representation format and user goal; within-participants factors were dining space status and layout orientation. To test hypothesis 3 all eight apartment ratings were included (the four apartments were rated twice) as well as attribute knowledge and whether the rating was produced before or after the attribute knowledge task, as additional independent variables. Six respondents had missing scores for the last apartment rating, these were hence excluded from the analysis for hypothesis 3.

### Manipulation checks

Prior to indicating their overall rating of apartment alternatives, participants in the user goal manipulation of entertaining guests were asked to rate how well the apartment could accommodate dinner parties for 6. A significant main effect for dining space status was found as expected, with apartments that featured a dedicated dining space rating higher (*M* = 3.89, SD = 1.10) than apartments without a dining space (*M* = 1.73, SD = 1.09; *F* (1, 206) = 777.55, *p* < .001, *η*^2^_*p*_ = 0.791). They were also asked to rate how well each apartment could accommodate having friends for a sleepover. As expected, a significant main effect for dining space status was also found for this dependent variable. Apartments with a dedicated dining space were rated higher (*M* = 2.94, SD = 1.02) than apartments without a dining space (*M* = 2.33, SD = 0.94;* F* (1, 204) = 129.57, *p* < .001, *η*^2^_*p*_ = 0.388). These manipulation check questions thus provide evidence of the user goal manipulation being successful for the entertaining guests condition.

Participants in the sunny disposition user goal manipulation rated what chance each apartment had of being naturally warm in winter. To assess the options for winter warmth, it was expected that participants would consider the orientation of the apartment’s layout in reference to north. A significant main effect for layout orientation was found as expected. Apartments that were north-facing rated higher (*M* = 3.26, SD = 1.14) than those facing south (*M* = 2.26, SD = 1.06; * F* (1, 208) = 95.65, *p* < .001, *η*^2^_*p*_ = 0.315). Participants were also asked to rate how well each apartment could accommodate drying clothes indoor on a clothes airer. For apartments that that were north facing, their capacity to dry clothes indoor was rated higher (*M* = 2.89, SD = 1.14) than those facing south (*M* = 2.36, SD = 1.02; * F* (1, 208) = 53.52, *p* < .001, *η*^2^_*p*_ = 0.205). These effects show that participants rated each apartment’s capacity to provide natural winter warmth and to dry clothes indoors by considering the orientation of the apartment alternatives and therefore the manipulation for the sunny disposition user goal condition can be considered successful.

### Representation format

It was expected that prior to familiarising respondents about the nature and benefits of the layout attributes (the attribute knowledge manipulation), the text formats would show a greater effect of the attributes on the respondent’s rating of the apartments than the floor plan formats (H1). Generally, the attribute effects were indeed larger in the text format condition than in the floor plan condition.

**Fig. 3:**
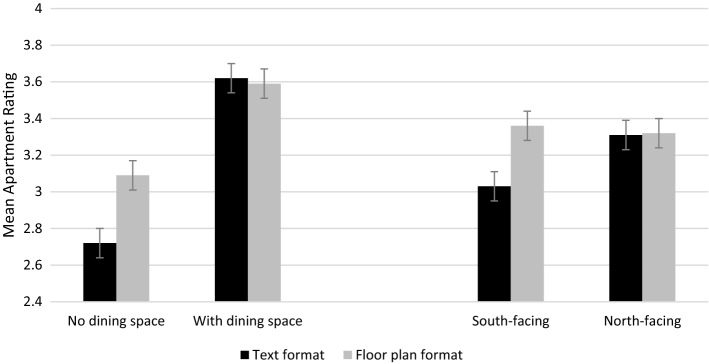
Interaction effect of representation format and attributes on apartment ratings (estimated marginal means, error bars ± 2 s.e.m.)

Across the conditions, the main effect of the dining space status attribute was significant. The mean apartment rating increased from *M* = 2.90 (SD = 0.78) for apartments without a dining space to *M* = 3.61 (SD = 0.75) for apartments with a dedicated dining space (*F* (1, 413) = 433.53, *p* < .001, *η*^2^_*p*_ = 0.512). When looking at the interaction of dining space status and representation format, this interaction was also significant (F (1, 413) = 39.90, *p* < .001, *η*^2^_*p*_ = 0.086). As Fig. 3 indicates, the effect of dining space status was larger for the text format than for the floor plan format with respondents showing more sensitivity to the dining space status attribute in the text format condition. The mean score for the text format of layouts with no dining space was *M* = 2.72 (SD = 0.77) versus *M* = 3.62 (SD = 0.77) for apartments with a dedicated dining space. This difference was smaller in the floor plan format, which scored *M* = 3.09 (SD = 0.74) and *M* = 3.59 (SD = 0.73), respectively. As demonstrated in Fig. 3, the mean apartment rating was similar in the text and floor plan format for apartments that had a designated dining space, but for apartments without a dining space, text formats scored significantly lower than floor plans.

North-facing apartments were expected to be more desirable than south-facing as in Australia northern sun provides direct sunlight and natural warmth in winter. The mean score for south-facing layouts was *M* = 3.19 (SD = 0.84) and north-facing was *M* = 3.31 (SD = 0.84), which is significant (*F* (1, 413) = 16.13, *p* < .001, *η*^2^_*p*_ = 0.038). When testing the effects of representation format and layout orientation, their interaction was significant, (*F* (1, 413) = 55.32, *p* < .001, *η*^2^_*p*_ = 0.118). In the text condition, the south-facing (*M* = 3.03, *SD* = 0.87) scored lower than the north-facing layouts (*M* = 3.31, SD = 0.90), as shown in Fig. 3. By contrast, in the floor plan condition, the south-facing layouts scored a higher mean (*M* = 3.36, SD = 0.78) than the north-facing layouts (*M* = 3.32, SD = 0.79), which implies that varying the orientation of apartments was not comprehended on the floor plan formats. Note these are the effects prior to the attribute knowledge manipulation.

Overall, these results show the effect for the floor plan format is smaller than for the text format for both layout attributes. H1 is therefore supported. Further, as shown in Fig. 3, the effect of layout orientation, was comparatively smaller than that of dining space status, implying that apartments featuring a dedicated dining space are more important to participants than north-facing apartments.

Interestingly however, the main effect for representation format was also significant (*F* (1, 413) = 16.55, *p* = < 0.001, *η*^2^_*p*_ = 0.039), with floor plans having a higher mean apartment rating (*M* = 3.34, SD = 0.78) than text formats (*M* = 3.17, SD = 0.89). This implies that floor plan representations have greater appeal even though understanding the spatial implications of varying layouts was more difficult to comprehend compared to layouts described in text.

### User goal

It was expected that the attributes would have greater effects on apartment ratings when they matched the assigned user goal condition (H2a) and that this effect will be greater in the floor plan format than the text format (H2b). We therefore tested the two and three-way interactions of user goal, representation format, and layout attributes, again using the observations for the four apartments (prior to attribute knowledge manipulation) only. There was no significant main effect for user goal (*F* (1, 413) = 0.783, *p* < .37) but there were significant interaction effects of user goal with both dining space status (*F* (1, 413) = 38.90, *p* < .001, *η*^2^_*p*_ = 0.086) and layout orientation (*F* (1, 413) = 19.71, *p* < .001, *η*^2^_*p*_ = 0.046), and also for representation format with each of dining space status (*F* (1, 413) = 39.08, *p* < .001, *η*^2^_*p*_ = 0.0.86) and layout orientation (*F* (1, 413) = 55.32, *p* < .001, *η*^2^_*p*_ = 0.118). Two three-way interactions were significant: the effect of user goal by representation format by layout orientation (*F* (1, 413) = 14.46, *p* < .001, *η*^2^_*p*_ = 0.034) and the effect of user goal by representation format by dining space status (*F* (1, 413) = 5.60, *p* = .018, *η*^2^_*p*_ = 0.013). We will now discuss the nature of these effects.

**Fig. 4:**
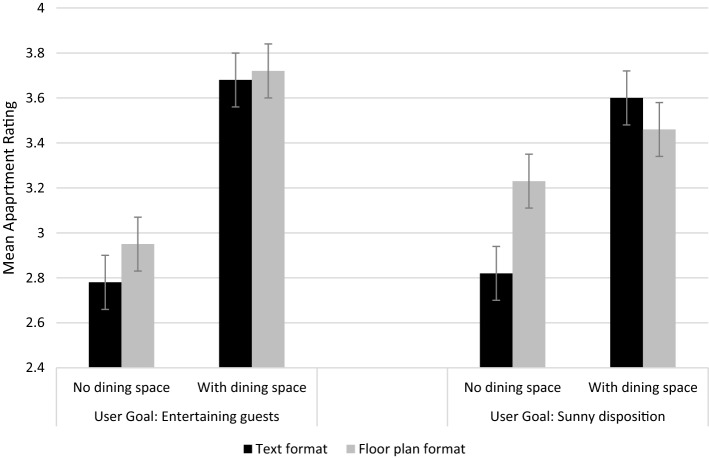
Interaction effect of user goal, representation format and dining space status on apartment ratings (estimated marginal means, error bars ± 2 s.e.m.)

To elaborate on the effects for the dining space status attribute, where the participant was assigned to the entertaining guests user goal condition, a significant effect was found on dining space status in the direction expected (*M*_nodining_ = 2.78, SD = 0.77 vs. *M*_dining_ = 3.68, SD = 0.75). Further, for the dining space status attribute (Fig. 4), where the participant was assigned to the entertaining guests user goal condition, and to the floor plan format group, there was a significant effect of dining space status in the direction expected (*M*_nodining_= 2.95, SD = 0.75 vs. *M*_dining_= 3.72, SD = 0.71). When comparing Figs. 3 and 4, it is evident that when assessing dining space status, articulation of preferences using floor plans was more sensitive when the respondents were in the entertaining guests user goal condition. However, the difference observed for floor plans was smaller than the difference observed for text formats (*M*_nodining_= 2.61, SD = 0.76 vs.* M*_dining_= 3.64, *SD* = 0.77). When examining the means for dining space status from respondents in the unrelated user goal group (sunny disposition), a significant effect was evident in the text format condition (*M*_nodining_= 2.82, SD = 0.77 vs. *M*_dining_= 3.60, SD = 0.77); however this effect was smaller than when the goal related to the attribute (entertaining guests). No significant effect was found for the floor plan format using data from participants in the unrelated (entertaining guests) user goal group (*M*_nodining_= 3.23, SD = 0.71 vs. *M*_dining_= 3.46, SD = 0.74). So, while the effects on dining space were larger when they were assessed in the entertaining guests user goal manipulation (H2a supported), this effect was not greater in the floor plan format than the text format, and therefore H2b is not supported for the dining space status attribute.

**Fig. 5:**
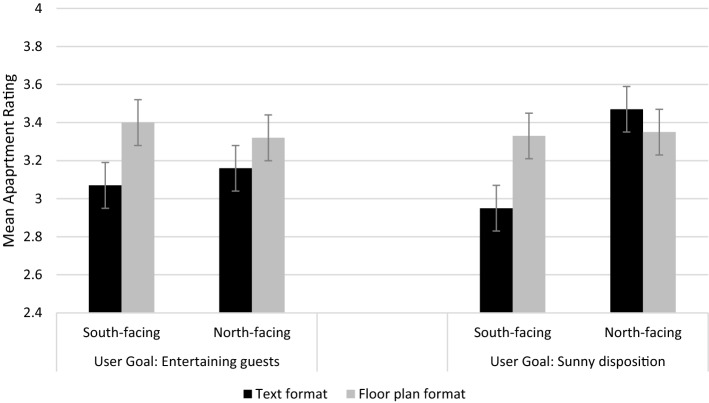
Interaction effect of user goal, representation format and layout orientation on apartment ratings (estimated marginal means, error bars ± 2 s.e.m.)

For the layout orientation attribute, where the participant was in the sunny disposition user goal condition, there was a difference in the direction expected (*M*_south_ = 3.14, SD = 0.80 vs. *M*_north_= 3.41, SD = 0.80). However, as shown in Fig. 5, where the participant was in the sunny disposition condition and floor plan format group, the means were virtually the same (*M*_south_= 3.33, SD = 0.75 and *M*_north_= 3.35, SD = 0.75). By comparison, in the text condition, north-facing layouts scored higher than south-facing layouts (*M*_south_= 2.95, SD = 0.79 vs. *M*_north_= 3.47, SD = 0.86). This implies that even when respondents were assigned to the sunny disposition user goal, layout orientation was not comprehended any better in the floor plan formats contrary to expectation (H2b rejected). In contrast, in the entertaining guests user goal condition the layout orientation effect was small (*M*_south_= 3.11, SD = 0.94 vs. *M*_north_= 3.14, SD = 0.90). Summing up, for layout orientation, attribute effects were larger when respondents were in the sunny disposition user goal group (H2a supported); however they were not larger in floor plan formats, than in the text formats (H2b rejected). Therefore, for both attributes dining space status and layout orientation, importance increased where participants were in the related user goal (entertaining guests and sunny disposition, respectively; H2a supported) yet this increased importance was not greater in the floor plan format; therefore H2b is not supported. Counter to expectation, and further supporting H1b, Figs. 4 and 5 demonstrate that the text format has a consistently larger effect on attribute preferences in the related user goal condition.

### Attribute knowledge

After evaluating apartment alternatives, participants were given a knowledge task about one of the two attributes before re-evaluating apartment alternatives. When they received information related to layout orientation (dining space status) it was expected that this attribute would reveal a greater effect on apartment preferences (H3a) and that this effect would be more prominent in floor plan formats than text formats (H3b). Analyses revealed an overall increase in the effect on apartment rating after the attribute knowledge task for dining space status (*F* (1, 403) = 9.24, *p* = .003, *η*^2^_*p*_ = 0.022) but no other knowledge-related effects were observed for dining space status. For layout orientation, the effect depended on whether the knowledge task related to the attribute (*F* (1, 403) = 11.82, *p* = .001, *η*^2^_*p*_ = 0.028). However, although there was an effect of representation (*F* (1, 403) = 6.56, *p* = .011, *η*^2^_*p*_ = 0.016), no significant interaction of representation and attribute knowledge was found.

**Fig. 6:**
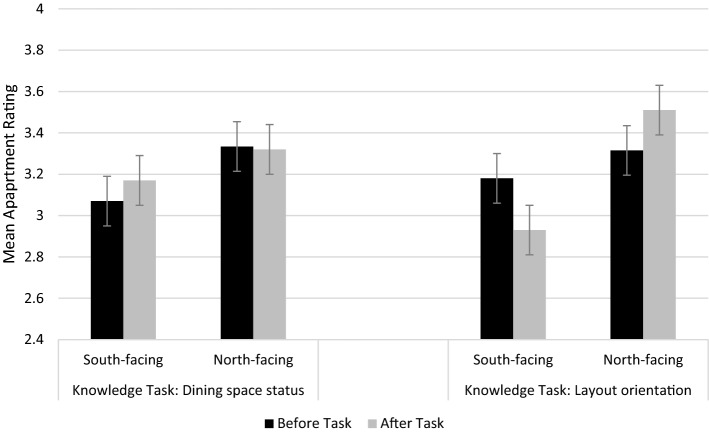
Interaction effect of attribute knowledge and layout orientation on apartment ratings before and after attribute knowledge task (estimated marginal means, error bars ± 2 s.e.m.)

The moderating effect of attribute knowledge task content (where the knowledge task related to the attribute) on layout orientation is visualised in Fig. 6. After reading information about the nature and benefits of layout orientation, participants scored layout orientation higher (*M* = 3.51, SD = 0.77) for north-facing than for south-facing apartments (*M* = 2.93, SD = 0.77). By comparison, when the provided information was unrelated to layout orientation (where knowledge content focused on dining space status), north-facing and south-facing apartments were more similar (*M* = 3.32, SD = 0.85, and *M* = 3.17, SD = 0.83). So, in the unrelated information condition there was little to no change from the first round of apartment evaluations, however where the content of the knowledge task was relevant to the orientation attribute (where knowledge content focused on layout orientation), respondents’ apartment ratings for layout orientation increased. This supports H3a. The lack of a significant interaction with representation format however means that H3b is rejected.

## Conclusions and discussion

### Main findings

The current study contributes to understanding tenant preferences for residential apartments and is especially applicable to the BTR tenant sub-class and therefore highly relevant to BTR landlords who typically own vast numbers of apartments. It is in their financial interest to target their product to the asset-light BTR renter demographic (Delahunty, 2022). The BTR asset class is well-established in Europe and North America and emerging in other anglophone liberal welfare states (Nethercote, 2020), as the world becomes more urbanised and owning a home becomes more out-of-reach for the Gen Z and Millennial generational cohorts. This is the first BTR tenant sub-class preference study in the SP housing preference and choice literature. Although only two attributes were measured, this study is a first step in understanding more about BTR tenant preferences.

Several scholars have noted that representation of spatial attributes such as building layout attributes are difficult to represent verbally (e.g. Jansen et al., 2009; Morrow-Jones et al., 2004; Oppewal & Klabbers, 2003; Orzechowski et al., 2005, 2012) and floor plan formats have not yet been tested in the SP literature. We address this gap with this study by comparing traditional verbal or text-based formats with floor plan formats.

Most precedent SP studies that compare the presentation style of housing-related alternatives, demonstrated that visual formats perform similarly to, or outperformed, verbal formats in rating tasks (Orzechowski et al., 2005; Jansen et al., 2009, 2011; Patterson et al., 2017). The current study does not support the findings of these studies, demonstrating quite the reverse. Lay-individuals, limited in their ability to read and comprehend floor plans (Ishikawa et al., 2011), showed greater sensitivity to layout variations represented by text formats than floor plan formats.

The overall finding that apartment tenants prioritised a dedicated dining space over apartment orientation was not surprising as Gao et al. (2013)’s study found that dining, and utility generally, is prioritised for smaller dwellings and attributes such as orientation are more important to tenants of medium sized dwellings. The benefit of being able to compare the rating of orientation on floor plan and text-based formats in the current study allows us to extend Gao et al. (2013)’s insights by demonstrating that comprehension of variations of orientation in floor plans was very poor. Although not measured in this study, future studies could compare the difference in comprehension and prioritisation of orientation amongst experts and lay-individuals for small and medium sized dwellings and on floor plan and text-based formats, building on the work of Boumová and Zdráhalová (2016), to demonstrate experts use a broader range of categories to evaluate layouts than lay-individuals.

It was expected that the layout attributes would have greater effects on apartment preference when respondents were focused on finding evidence that their assigned user goal was being met. Such an effect was indeed found; however this effect was not larger in the floor plan format than the text format as expected. We therefore conclude that despite individuals’ tendency to prioritise attributes related to their goals (Vischer, 1985) they did not pay particular attention to the feature on floor plans that related to their assigned goal condition and showed no evidence of improved comprehension of floor plans with a goal to focus on.

It was also expected that the effects of the two attributes on apartment preferences would increase after participants undertook an attribute knowledge task relevant to the attribute, especially in the floor plan format. No effect was found of attribute knowledge on the dining status attribute, yet a significant effect was found on the orientation attribute after the orientation-related knowledge task was undertaken, showing an increase in apartment ratings. However, while the manipulation was successful, surprisingly, no influence was found of attribute knowledge on the effect of representation format.

Although the current study does not dispute Gao et al. (2013)’s findings that apartment orientation is less important to tenants of small dwellings than utility attributes, this finding demonstrates that with the benefit of an attribute knowledge task, small well-oriented apartments do yield increased ratings, however not to the extent that they outperform ratings of utility attributes. Building on this finding, and those of Gao et al. (2013) and Boumová and Zdráhalová (2016), a future study with a sample of varying levels of experts (for example individuals that have recently moved home, valuers, real estate agents, builders and architects) and lay-individuals could be undertaken to demonstrate to what extent experts are trained to understand the implications of a well-oriented apartment (over and above information provided in the attribute knowledge task) including the effect on thermal comfort, indoor clothes drying, health and well-being, and utility bills.

Remarkably, the findings showed a main effect of representation format, demonstrating that overall, respondents rated apartments represented by floor plan formats higher than those represented by text formats. This implies that although the lay-individual may have difficulty reading and comprehending floor plans, apartment information that includes a floor plan potentially improves its rating. This supports Montañana and Llinares’ (2015) findings that highlighted layout features positively influence feelings and emotions that affect purchase decisions in the context of property developers' promotions

In summary, although floor plan representations were rated higher than text descriptions overall, our findings show that we should be careful not to over-estimate the ability of floor plan formats to convey information to lay-individuals better than text formats. We did find that attributes supported by a relevant user goal, increased their importance; however this increase was not seen in the floor plan formats. Further, we expected that there would be an effect of attribute knowledge depending on whether the knowledge task was related or unrelated to the user goal condition and the layout attribute. There was however only an effect of increased importance for layout orientation and this effect was not moderated, neither by representation format nor by the user goal manipulation.

Based on these findings, it is important to provide floor plans in combination with other verbal and visual representation formats when describing property attributes to lay-individuals. Although floor plans appear to have a limited effect on comprehension of apartment features, apartment descriptions that include floor plans are rated more highly than those without, indicating they are important to respondents when considering apartment layout options and justifying them as a useful tool in SP methods.

### Limitations and future research

Limitations to this study include firstly the sample composition. Most of the sample was between 18 and 20 years old and not experienced or familiar with reading floor plans. Although the study contributes to the literature on the legibility of floor plans to the lay-individual, variation of experience and the effect of spatial ability amongst lay-individuals were not measured.

A second limitation is that the study manipulated only two spatial attributes. These attributes however are relevant in the context of apartment evaluations and the findings thus do reveal a relevant insight about how spatial features can be represented to prospective users. There are to our knowledge no studies that compare effects of housing layout attributes floor plan formats and text formats. Our contribution exists not only through its empirical findings but also by demonstrating an approach to the systematic study of such effects.

Thirdly, the authors acknowledge that content equivalence of the stimuli is impossible to achieve as text draws attention to attributes and floor plans diagrammatically present a combination of attributes focal to the study as well as non-systematically varied attributes thereby rendering it difficult to control which attributes individuals will pay attention to.

Future studies other than extensions to the current study and those of Gao et al. (2013) and Boumová & Zdráhalová (2016), mentioned in the previous section, could build on Montañana and Llinares’ (2015) findings that highlighted layout features positively influence feelings and emotions that affect purchase decisions in the context of property developers' promotions. While this study shows that apartments presented as floor plans are rated higher than those presented in text formats, a follow-up study is needed to better understand why people perceive floor plans to be a benefit as this was not captured in this study. Individuals appear to value their availability, suggesting they integrate floor plan information with information presented in other formats to assess how they would feel about spaces if they were to use them in the future.

Future studies could consider modifying the context of this research to test floor plan stimuli on the broader residential asset class and range of property types and end users such as owner occupiers and short-term tenants. Additionally, this study could be extended to other property asset classes such as commercial, including office, retail and hotels where preferences for anchor tenants, lessees and visitors could help developers to tailor the architectural design to the desires of the end user. At different scales, future studies could look at effects of design features on representation formats such as site plans, master plans and indoor maps. In sum, while this study presents only a first step in understanding the role of floor plans, it shows that floor plans are less self-evident than experts may think.

## Data Availability

The datasets generated during and/or analysed during the current study are available from the corresponding author on reasonable request.

## Footnotes

1 Alternatively, individuals choose from designed sets of multi-attribute alternatives, the approach is then typically referred to as discrete choice experiments.
